# Bioactivity of *Ribes nigrum* L. Juice and Waste Extracts: Chemical Composition, Antioxidant, and Antiproliferative Properties

**DOI:** 10.3390/plants15030356

**Published:** 2026-01-23

**Authors:** Milica Trajković, Bojana Miladinović, Dragan Mihailović, Stevo Najman, Milica Milutinović, Milica Randjelović, Miloš Jovanović, Nemanja Kitić, Katarina Šavikin, Dušanka Kitić

**Affiliations:** 1 Department of Pharmacy, Faculty of Medicine, University of Niš, Blvd. Dr Zorana Djindjića 81, 18108 Niš, Serbia; milica.trajkovic@medfak.ni.ac.rs (M.T.); milica.milutinovic@medfak.ni.ac.rs (M.M.); milica.randjelović@medfak.ni.ac.rs (M.R.); milos.jovanovic@medfak.ni.ac.rs (M.J.); 2 Human Polyclinic, Petra Seratlića 21, 18108 Niš, Serbia; draganmihailovic54@gmail.com; 3 Department of Biology and Human Genetics, Faculty of Medicine, University of Niš, Blvd. Dr Zorana Djindjića 81, 18108 Niš, Serbia; stevo.najman@medfak.ni.ac.rs; 4 Scientific Research Center for Biomedicine, Faculty of Medicine, University of Niš, Blvd. Dr Zorana Djindjica 81, 18108 Nis, Serbia; 5 Department of Physiology, Faculty of Medicine, University of Niš, Blvd. Dr Zorana Djindjića 81, 18108 Niš, Serbia; voidruner@gmail.com; 6 Institute for Medicinal Plants Research “Dr. Josif Pančić”, Tadeuša Košćuškog 1, 11000 Belgrade, Serbia; ksavikin@mocbilja.rs

**Keywords:** anthocyanins, black currant fruit, Ki67 index, histopathology, kidney, polyphenolic compounds, vitamin C

## Abstract

This study aimed to assess phytochemical profiles and antioxidant activities of lyophilized black currant fruit juice (BCLJ) and its corresponding waste extract (BCLW) from the Čačanska crna variety, and to evaluate their antiproliferative properties. The main anthocyanins quantified through HPLC-DAD analysis were delphinidin-3-*O*-rutinoside and cyanidin-3-*O*-rutinoside, with significantly higher levels in BCLW. Antioxidant activity was examined using the DPPH and β-carotene/linoleic acid methods, with BCLW showing superior effects in both. Antiproliferative potential was evaluated by determining the Ki67 index in renal epithelial cells of rats treated with BCLJ or BCLW. Thirty healthy male rats were randomly assigned to five groups (n = 6) and administered BCLJ or BCLW orally for ten days, receiving 100, 200, and 300 mg/kg b.w. of BCLW (BCLW1, BCLW2, and BCLW3 groups, respectively) or 200 mg of BCLJ. Histopathological and immunohistochemical parameters were assessed in rats’ kidneys. Across all epithelial types (cortical proximal tubules, distal medullary proximal tubules, collecting ducts, and urothelial cells of the renal pelvis), the highest Ki67 indices were observed in control animals, particularly in collecting ducts and cortical proximal tubules. The lowest Ki67 values in cortical proximal tubules occurred in the BCLW2 group (*p* < 0.05 vs. control). These findings suggest that black currant preparations could be valuable functional ingredients.

## 1. Introduction

Black currants (*Ribes nigrum* L., Grossulariaceae) are berries primarily cultivated in Northern Europe, parts of North America, and Northern Asia. This plant grows as a small shrub typically to a height of 1 to 2 m and produces small dark-purple fruits [1,2].

Black currants are abundant in bioactive compounds, including various vitamins, polyunsaturated fatty acids, and polyphenolic substances [3,4,5,6,7]. Among polyphenols, the predominant are anthocyanins (3-*O*-glycosides and 3-*O*-rutinosides of delphinidin and cyanidin), making up to 85% of the total phenolic content in different varieties of black currants [4,5]. Beside them, there are also flavonols (kaempferol, quercetin, and myricetin), phenolic carboxylic acids (caffeic, chlorogenic, and ferulic acid), and tannins [6,7].

Black currant products have been extensively researched for their multiple health effects. Extracts from black currant berries exhibit antioxidant activity, along with antiproliferative, anti-inflammatory, and cardioprotective effects [8,9,10,11]. The antioxidant activity largely depends on their anthocyanin content, primarily derived from delphinidins, while cyanidins contribute to a lesser extent [12]. Antiproliferative and antioxidant properties of anthocyanin rich extracts from black currants have been investigated mostly in vitro. In vitro studies have primarily investigated the antiproliferative and antioxidant properties of anthocyanin-rich extracts from black currants. For instance, Diaconeasa et al. [9] studied the antiproliferative activity of anthocyanin-rich fractions derived from commercially available black currant juice on three tumor cell lines: HeLa, A2780, and B16F10. They found that glycosylated anthocyanins extracted from black currant juice led to a dose-dependent reduction in cell viability, with IC50 values of 281 μg/mL for HeLa cells, 259.8 μg/mL for A2780 cells, and 224 μg/mL for B16F10 cells. Additionally, Horie et al. [10] investigated the effects of a diet supplemented with 3% black currant extract in ovariectomized (OVX) rats, which serve as models for menopause. The study focused on elastin degradation and the prevention of pathological vascular remodeling during this stage. They observed that elastin breakdown in abdominal aorta tissue decreased by 21.4% in the OVX black currant extract group, compared to 35% in the OVX control group. Furthermore, a study by Miladinovic et al. [8] demonstrated strong antiproliferative activity of delphinidin-3-*O*-rutinoside against NCI-N87 (gastric) and Caco-2 (intestinal) cancer cells in vitro, with IC50 values of 24.9 µM and 102.5 µM, respectively. Black currant berry extract also showed significant inhibition of myeloperoxidase (MPO) activity, with an EC50 value of 14.4 µg/mL. MPO is an enzyme that can cause substantial damage to macromolecules in cell membranes during acute and chronic inflammation. This suggests that black currant extract may offer potential benefits in combating inflammatory and cardiovascular diseases. Black currant berry extract (EC50 = 14.4 µg/mL) has been shown to strongly inhibit the of myeloperoxidase (MPO) activity, an enzyme that can cause significant damage to macromolecules in cell membranes under certain conditions of acute and chronic inflammation [11]. This suggests that black currant extract may offer potential benefits in combating inflammatory and cardiovascular diseases. Flavonols such as quercetin, myricetin, and isorhamnetin also play a beneficial role in antioxidant action [13].

The mechanisms underlying carcinogenesis, diseases caused by oxidative stress, and aging (cardiovascular diseases, chronic obstructive pulmonary disease, chronic kidney disease, and neurodegenerative diseases), are complex and involve multiple regulatory levels [14,15]. For instance, the production of reactive oxygen species (ROS) can damage cellular macromolecules. Moreover, mutations in critical genes that arise after the bioactivation of these molecules can adversely impact cell proliferation. Both ROS and insufficient DNA repair can result in gene damage and mutations, which are significant internal mechanisms in the development of diseases [14].

The influence of anthocyanins on the development of the aforementioned diseases varies significantly. Delphinidin and its derivatives, which are the main anthocyanins in black currants, contain hydroxyl groups that are crucial for their ability to scavenge free radicals associated with the progression of diseases caused by oxidative stress. By reducing oxidative stress, anthocyanins, specifically delphinidin, can interfere with both the initiation and promotion stages of carcinogenesis [16]. Additionally, delphinidin induces apoptosis and causes cell-cycle arrest in cancer cells through pathways involving mitochondrial signaling and caspase activation. It can halt the cell cycle at the G2/M phase by modulating the expression of cyclins and cyclin-dependent kinases (CDKs). Furthermore, delphinidin suppresses angiogenesis by downregulating pro-angiogenic factors, such as VEGF. Its antioxidant and anti-inflammatory properties support these anti-angiogenic effects, limiting the nutrient and oxygen supply to tumor cells [16]. Moreover, delphinidin exhibits anti-inflammatory activity by inhibiting key inflammatory mediators including NF-κB and COX-2, which are often upregulated in cancer cells. This suppression of inflammatory signaling contributes to its anticancer effects, as delphinidin also modulates pathways involved in cell proliferation, tumor growth, and metastasis [17]. According to Nanashima et al. [18], black currant extracts that are rich in anthocyanins were found to reduce the number of cells in the S and G2/M phases while increasing the number of cells in the G0/G1 phase in the MCF10A line of healthy human breast epithelial cells. The anthocyanin-rich black currant extract may inhibit the signaling pathways related to gene expression. Potential mechanisms for this effect may involve the regulatory roles of polo-like kinase in mitosis and cell death. Research conducted by Miladinovic et al. [8] demonstrated good antiproliferative activity in vitro on NCI-N87 (gastric) and Caco-2 (intestinal) malignant cells.

In daily pathological practice, Ki67 (Kiel 67) is an accepted marker of cell proliferation. An increase in Ki67 expression indicates a higher level of cell proliferation [19,20]. Ki67 is a nuclear non-histone protein found in proliferating cells. Cell proliferation occurs during all phases of the cell cycle except for G0 and early G1, with the highest levels observed during the G2 and M phases. In healthy rat kidneys, the greatest number of Ki67 positive cells is typically found in the upper medulla of the kidney [21].

Therefore, the present research aimed to investigate the types and content of secondary metabolites found in the black currant variety Čačanska crna lyophilized fruit juice (BCLJ) and waste extract (BCLW). Additionally, we sought to determine the Ki67 index in normal adult rat kidney epithelial cells treated with both the black currant juice and the waste extract. Since the effects of black currant extracts on normal kidney tubular epithelial cells have not yet been reported, our study may provide valuable insights into the potential effect of BCLJ and BCLW in vivo.

## 2. Results

### 2.1. Chemical Composition of Lyophylized Black Currant Juice (BCLJ) and Waste Extract (BCLW)

The chemical composition includes the content of total polyphenols, total tannins, and flavonols, as well as the HPLC determination of ascorbic acid (vitamin C) and anthocyanins. The results indicate the type and quantity of polyphenolic compounds present in the black currant samples, revealing that both BCLJ and BCLW are significant sources of these biomolecules. Notably, BCLW contained higher levels of all the anthocyanins analyzed compared to BCLJ. Conversely, the total amount of ascorbic acid was 15 times greater in BCLJ. Additionally, the total polyphenol and tannin content was also more pronounced in BCLJ. Among the individual compounds, D3R emerged as the most prominent anthocyanin found in both BCLJ and BCLW. Myricetin was the predominant flavonol in both samples, while the quercetin content was found to be 20 times higher in BCLJ than in BCLW. The chemical composition of BCLJ and BCLW is detailed in Table 1.

### 2.2. Antioxidant Activity

This activity was assessed using two different in vitro laboratory methods. The first method utilized the DPPH reagent, which measures the potential of the samples to sequester free radicals through a hydrogen atom-donating process. The BCLJ sample required a higher concentration to effectively scavenge the DPPH radical, with a measurement of 4.89 mg/mL, indicating lower antioxidant activity compared to BCLW, which demonstrated a better antioxidant capacity at 3.97 mg/mL. The antioxidant activity of the samples is summarized in Table 2. Butylhydroxytoluene (BHT), butylhydroxyanisole (BHA), ascorbic acid (AsA), and Trolox demonstrated more potent radical scavenging activities than the samples that were tested, with IC50 values of 22.82 ± 2.07, 2.44 ± 0.09, 6.15 ± 0.64, and 4.74 ± 0.33 µg/mL, respectively.

The second method employed was the β-carotene/linoleic acid bleaching model, which evaluates the ability of the preparations to prevent lipid peroxidation. In this system, BCLW again showed superior antioxidant activity, measuring 1.32 mg/mL. In comparison, BHT, BHA, AsA, and Trolox demonstrated superior antioxidative activities compared to the tested samples (IC50 [µg/mL] = 0.03 ± 0.00, 0.04 ± 0.01, 1.69 ± 0.11, and 22.95 ± 1.52, respectively).

### 2.3. Antiproliferative Activity Expressed in Adult Rat Kidney Epithelial Cells

In all types of kidney epithelia, the highest Ki67 index values were observed in the control group, specifically in the collecting ducts (3.3 ± 0.33%, *p* < 0.01, compared to all other groups) and proximal cortical tubules (2.93 ± 0.37%). The difference between these two values was not statistically significant.

The lowest Ki67 index values were observed in the BCLW2 group in all types of kidney epithelia.

**Figure 1:**
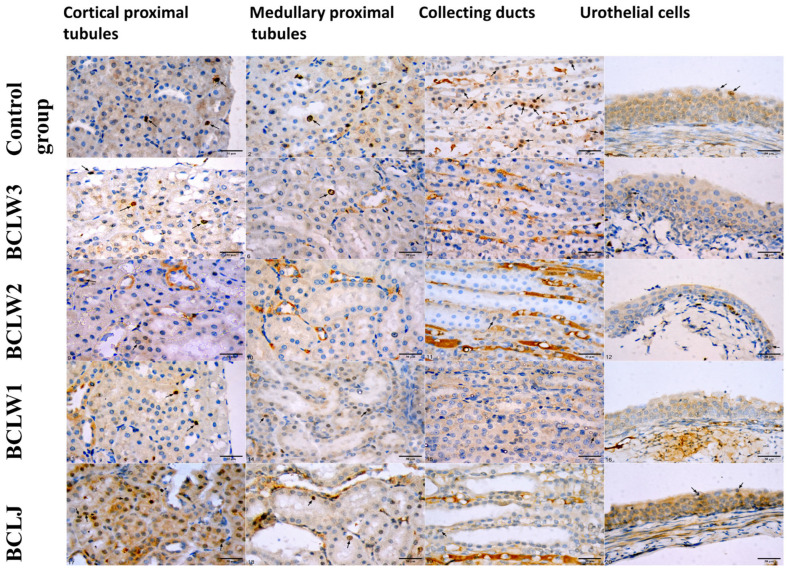
Ki67 immunohistochemistry in kidney epithelial cells of cortical proximal tubules, proximal tubules of distal medulla, collecting ducts, and urothelial cells. Arrows indicated Ki67 positive cell nuclei. Obj. ×40, scale bar equals 30 µm. BCLW E1 = black currant waste extract 100 mg/kg bw; BCLW E2 = black currant waste extract 200 mg/kg bw; BCLW E3 = black currant waste extract 300 mg/kg bw. BCLJ = black currant juice 200 mg/kg.

**Figure 2:**
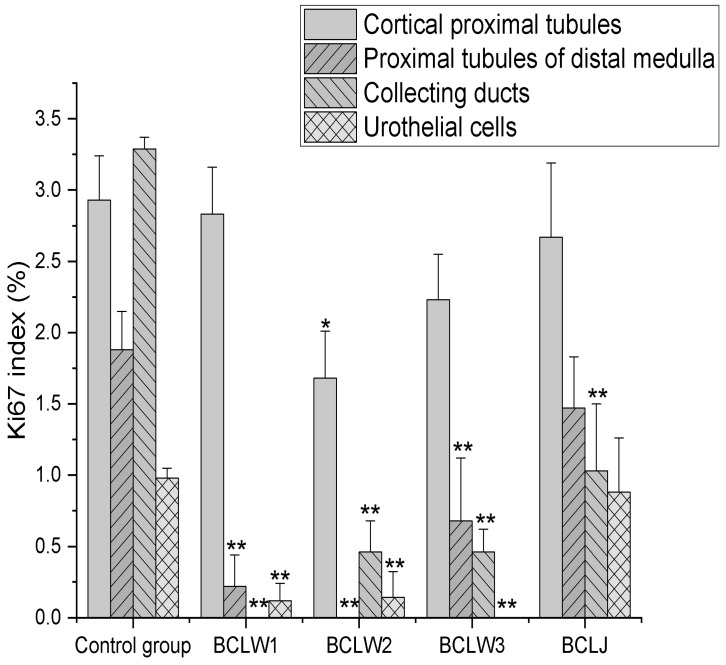
Ki67 index of adult rat kidney epithelial cells. Bars indicate the standard error of the mean value. Significance levels are denoted as follows: * *p* < 0.05; ** *p* < 0.01, compared to control group. BCLW E1 = black currant waste extract 100 mg/kg bw; BCLW E2 = black currant waste extract 200 mg/kg bw; BCLW E3 = black currant waste extract 300 mg/kg bw; BCLJ = black currant juice 200 mg/kg bw.

Ki67 positivity was not found in collecting ducts of the BCLW1 group, proximal tubules of the distal medulla of the BCLW2 group, or urothelial cells of the renal pelvis of the BCLW3 group (Figure 1 and Figure 2).

The Ki67 index in collecting ducts in the BCLJ group was significantly lower than in the collecting ducts of the control group (*p* < 0.01). Other differences in the Ki67 index between the BCLJ and the control group were not statistically significant.

## 3. Discussion

Over the years, there has been a rising incidence of chronic non-infectious diseases, which pose a significant public health risk. As a result, efforts are being made to prevent these diseases and their related complications [22,23]. Berries are commonly used in both the prevention and treatment of various health issues [24]. Our study aimed to estimate the overall chemical composition and antioxidative activity of black currant and its preparations, as well as their antiproliferative effects. The total polyphenolic content was found to be 6.97 mg gallic acid equivalent per gram (GAE/g) for BCLJ and 4.54 mg GAE/g for BCLW. A slightly lower tannin content was observed (4.19 mg GAE/g) in BCJL and 3.98 mg GAE/g in BCLW. The polyphenolic content in our study is comparable to that in another study, which found the total phenolic content in black currant berry extract to be 15.0 mg GAE/g [25]. Additionally, that study indicated that the black currant variety Čačanska crna had the highest content of polyphenols and flavonoids among various cultivars. Our findings for this plant species showed similar characteristics.

Regarding the anthocyanins, D3R was found to be the most abundant in both BCLJ (394.92 μg/100 mg) and BCLW (757.78 μg/100 mg), with BCLW exhibiting a higher concentration of this compound. These findings are consistent with previous research. In a study by Djordjevic et al., *Ribes nigrum* L. variety Čačanska crna was identified as having D3R as the predominant anthocyanin in its juice [26]. The study also revealed that berries from 2-year-old shoots contained less D3R (243.0 mg/kg fresh weight) compared to those from 3-year-old shoots (310.2 mg/kg fresh weight). This suggests that the age of the shoots may significantly influence the fruit’s chemical composition. Another investigation also highlighted D3R as the most prevalent among four main individual anthocyanins [9]. In our study, the concentrations of C3R were measured at 271.13 μg/100 mg in BCLJ and 464.35 μg/100 mg in BCLW, indicating that rutinosides of delphinidin and cyanidin are particularly prominent, especially in BCLW. Djordjevic et al. noted that C3R levels were 225.3 mg/kg fresh weight in Čačanska crna berry extract from 2-year-old shoots [26]. Consistent with the present findings, a comprehensive analysis of the anthocyanin profiles of berries from ten black currant cultivars grown in Illinois (USA) demonstrated that D3R was the dominant anthocyanin across all investigated cultivars. Although the relative proportions of individual anthocyanins varied among cultivars, rutinoside-derived heterosides were consistently more abundant than their corresponding glucoside forms [27]. Similarly, a recent study evaluating eight black currant varieties cultivated in Serbia, including the variety Čačanska crna, showed that D3R was predominantly identified as the major anthocyanin, with rutinoside heterosides prevailing over glucosides in all analyzed samples [28]. This trend, with dominant rutinoside heterosides and D3R as the major individual anthocyanin, was also observed in extracts of black currant pomace from the UK [29]. Together, these findings indicate a high degree of consistency in the anthocyanin composition of black currant berries across different cultivars and growing regions.

Among flavonols, myricetin was the most prominent in both samples, with significantly higher quantities found in BCLJ. The levels of quercetin and kaempferol were also higher in BCLJ. In the aforementioned study on black currant pomace extracts from the UK, myricetin, kaempferol, and quercetin derivatives were also reported as the dominant flavonols. However, they were detected in the form of rutinoside and glucoside heterosides and were not observed in their aglycone forms [29]. Paunović et al. [25] reported 6.33 mg of myricetin per 100 g in the fresh fruit of Čačanska crna. However, the flavonol type and content differ when compared to other studies, where quercetin is often the most abundant flavonol. Paunović et al. indicated that quercetin is the most prominent compound in black currant ethanolic extract, with a value of 10.5 mg per 100 g. These differences may arise from various factors, as anthocyanin synthesis depends on plant species, environmental conditions, and genetic factors [26,30]. Besides regulatory and structural genes, multiple enzymes play a role in the synthesis process. Authors have suggested that ultraviolet-B radiation, weather conditions, and oxygen significantly impact the stability and type of flavonols [31,32].

Compared to other fruits, black currants have higher levels of ascorbic acid, and their content remains relatively stable due to a significant quantity of anthocyanins and polyphenols. These compounds play an important role in preserving ascorbic acid and maintaining its stability [26,33]. In our study, the total ascorbic acid content was measured at 750 μg/100 mg in BCLJ and 50.0 μg/100 mg in BCLW. Research conducted by Djordjević et al. compared ascorbic acid levels among multiple species of *Ribes nigrum*. The variety Čačanska crna exhibited the highest ascorbic acid content, with 175.3 mg per 100 g fresh weight (FW) in 2-year-old shoots and 158.33 mg per 100 g FW in 3-year-old shoots [26].

In comparison to other berries, such as *Ribes rubrum* L., our samples demonstrate a higher ascorbic acid content. Cvetković et al. reported that Redpoll lyophilized juice contained 230 mg of ascorbic acid per 100 g of lyophilized juice [34].

The antioxidative activity of black currant berries is one of their most significant pharmacological benefits. Due to their high levels of polyphenols, flavonols, and tannins, black currants are an excellent source of natural antioxidants [26,35,36]. In our research, we evaluated the antioxidant capacity using two in vitro models. According to the DPPH model, BCLW exhibited a higher antioxidant potential, indicated by a lesser depletion of the DPPH reagent. This increased antioxidant activity may be attributed to a larger amount of delphinidin and cyanidin glucosides and rutinosides present in the berries. Our findings differ from those of previous studies. Research conducted by Paunović et al. reported an IC50 (inhibitory concentration at 50%) value of 12.4 ± 0.93 mg ascorbic acid per gram of black currant ethanolic extract. According to their findings, the Čačanska crna variety demonstrated the highest antioxidant capacity among other varieties [25]. Numerous studies have indicated a positive correlation between total phenolic content and antioxidant activity, as evaluated using the DPPH model [37]. Flavonoids make up the majority of polyphenols and play a significant role in overall antioxidant capacity due to their chemical structure [21,38]. Their functional hydroxyl groups, along with their substitution patterns and configurations, contribute to the elimination of free radicals. Moreover, flavonoids can chelate metals, which helps prevent the formation of free oxygen radical [39]. Beyond the most commonly studied polyphenolics, recent studies have increasingly emphasized the role of black currant polysaccharides as contributors to antioxidant activity [40].

Since the majority of anthocyanins in BCLW were identified as D3R and C3R, we can conclude that these compounds significantly contribute to the antioxidative capacity of the investigated samples. These findings align with Azman et al. [29], who found that anthocyanins were the primary contributors to antioxidant activity in black currant pomace extracts, while total flavonols and hydroxycinnamic acids showed weaker correlations. Their ability to neutralize free oxygen radicals and break down peroxides plays a key role in enhancing the overall antioxidant capacity [41]. The antioxidant potential of anthocyanin-rich extracts of berries has been thoroughly investigated. Solcan et al. [24] demonstrated significant antioxidant activity in both lingonberry and black currant extracts. A dose-dependent reduction in reactive oxygen species (ROS) levels was observed. It has been described that delphinidins are more prominent as antioxidants in comparison to cyanidins [26].

Regarding the *β*-carotene/linoleic acid antioxidative activity in our study, BCLW showed greater antioxidant activity than BCLJ, as observed with DPPH. While BCLW consistently exhibited superior activity in both assays, differences were noted in the affinities of the two samples toward the aqueous and lipid phases. Indeed, BCLJ displayed lower IC50 values in the DPPH assay compared to the *β*-carotene/linoleic acid system, whereas the opposite trend was observed for BCLW. The better performance of BCLJ in the DPPH system likely reflects a preferential distribution of antioxidant constituents in the aqueous phase, which may be related to the higher content of total ascorbic acid in this sample, a well-known hydrophilic antioxidant. A similar trend, with lower IC50 values in DPPH compared to the *β*-carotene/linoleic acid system, was observed for all eight black currant varieties from Serbia in our previous study [28]. On the other hand, the higher antioxidant activity observed in the *β*-carotene/linoleic acid system reflects a greater affinity of BCLW bioactive compounds for lipid phases, which is understandable given that 60% ethanol was used as the extraction solvent and is a less polar medium than juice, an aqueous solution. To the best of our knowledge, there are no comparable literature findings on the topic of the *β*-carotene linoleic acid system in black currant lyophilizates.

The results of our study indicate that treatment with black currant extract significantly decreases the Ki67 index of kidney tubular epithelial cells and urothelial cells in the renal pelvis. This is shown as significant decrease in the Ki67 index. On the other hand, the significant effects of BCLJ are observed only in the collecting ducts. Upon examining rat kidney tissue, we found that the highest Ki67 index values were observed in all types of kidney epithelia (in the control group, in the cortical proximal tubules and the proximal tubules of the distal medulla). In contrast, the BCLW2 group exhibited the lowest Ki67 index values in these tubules, with results showing significance at *p* < 0.05 and *p* < 0.01 compared to the control group, respectively. The normal adult kidney has a very low rate of cell turnover, but it can respond quickly to injury by generating new cells to replace those that have died. Our results indicate two peaks in the Ki67 index: one in the collecting ducts and another in the cortical proximal tubules. It is possible that kidney embryology plays a significant role in these processes. The kidney of amniotes develops from two distinct progenitor cell populations derived from the intermediate mesenchyme: the ureteric bud and the metanephric mesenchyme. The ureteric bud is responsible for developing all the cell types that form the mature collecting ducts and the ureter. In contrast, the metanephric mesenchyme gives rise to all the cell types that make up the mature nephron, as well as some vascular and stromal components [42]. A previous study on the normal adult rat kidney found the highest number of Ki67-positive cells in the upper medulla [21]. Our results are in concordance with these finding, as we also observed the highest number of Ki67-positive cells in the collecting ducts.

In our study, the Ki67 index was estimated by manually identifying Ki67-positive and Ki67-negative nuclei of epithelial cells and expressing the results as a percentage of positive cells. This method is similar to those used in previous studies [21,43]. In other research, the Ki67 index was reported as the percentage of the Ki67-positive area relative to the total area of the microscopic field [44,45,46,47], mainly with higher Ki67 index values than in previous research.

## 4. Materials and Methods

### 4.1. Plant Material and Sample Preparation

Black currant (*Ribes nigrum* L.), variety Čačanska crna, was harvested and bred in Radmilovac, an experimental field of the Faculty of Agriculture University of Belgrade. Fully ripe berries were collected from the end of June until the beginning of July 2020. After the collection, the fruits were stored frozen at −18 °C. Firstly, the fruits were defrosted and pressed for squeezing fruit to obtain the juice. The juice was used for further analysis. The residue after straining (waste) was dried on filter paper for 24 h. Then, it was dried in a laboratory dryer at 40 °C for 48 h (Instrumentaria ST 01/02, Zagreb, Croatia). The waste was later ground in a mill (UMČ-20, Biljotehnika, Pančevo, Serbia). This shredded waste was afterwards griddled, in accordance with applicable regulations [48]. To extract the plant material, the selected method was maceration. Maceration was performed via 60% ethanol (material to solvent ratio was 1:20). Subsequently, maceration was performed using a laboratory shaker (Unimax 1010, Heidolph, Schwabach, Germany) with 170 rpm rotation for one hour at room temperature [34,49]. The liquid extracts were filtered in order to separate the residues. The solvent was then removed from the material by a rotary evaporator (Büchi AG, Uster, Switzerland). Preparations of juice and waste were used for the further lyophilization process. The waste extract was kept at −18 °C.

Freezing the juice and waste extracts at −80 °C was necessary. Afterwards, the ex-tracts were lyophilized at −60 °C (at a pressure of 0.011 mbar) for 24 h and at −60 °C (at a pressure of 0.0012 mbar) for one more hour. This was performed in order to remove the capillary water residues (Beta 1–8 Freeze Drier, Martin Christ, GmbH, Osteroide am Harz, Germany).

### 4.2. Determination of Total Polyphenols

Analysis of the total polyphenols was performed using the Folin–Ciocalteu (F–C) method, concordant with the method by Makkar [34,50]. Spectrophotometry was used to investigate the samples and standard, at an absorbance of 725 nm. Triplicate measurements were made for each sample. The results were noted by gallic acid equivalents (GAE) (mg GAE/g juice or extract).

### 4.3. Determination of Total Tannins

The quantity of total tannins was evaluated using the abovementioned Folin–Ciocalteu (F–C) [34,50]. The quantification of total tannins was performed with the F-C reagent and insoluble polyvinylpolypyrrolidone (PVPP). This molecule has the ability to attach tannins from the solution. The difference between total and non-tannic polyphenols represented the total amount of tannins. The results were noted as the same as per total polyphenols (mg GAE/g juice or extract).

### 4.4. Determination of Anthocyanins (AC)s

The quantification of ACs was conducted in accordance with the method by Cacace and Mazza, with minor updates [34,51]. Lyophilized juice and extract (10 mg) were diluted in 1 mL of deionized water, with the addition of sulfuric acid to pH 2.5. The sample was then inserted in an ultrasonic bath for 10 min. Afterwards, it was centrifuged at 4000× *g* rpm at room temperature. The separated supernatant was filtered through a Millipore membrane filter (0.45 μm). The separated supernatant was further used for HPLC analysis.

#### Chromatographic Analysis

Chromatography was performed using an Agilent 1200 system (Agilent Technologies, Palo Alto, CA, USA). This system had a diode array detector (DAD), an automatic sampler, and a control system included. Compounds were separated via Merck Purospher STAR RP-18e analytical column (150 mm × 4.6 mm i.d., 5 μm particle size; Merck, Darmstadt, Germany). Mobile phase A was Trifluoroacetic acid at a 0.1% concentration, whilst mobile phase B was acetonitrile. Isocratic conditions were set to perform the separation, and the flow rate was 1 mL/min at 25 °C. The volume of the sample was 20 μL. Firstly, the detection of AC was performed at 520 nm. The quantification of AC was performed by a calibration curve. It was made by standard solutions of ACs (delphinidine-3-*O*-rutinoside, delphinidine-3-*O*-glucoside, cyanidine-3-*O*-glucoside, and cyanidine-3-*O*-rutinoside). Five increasing concentrations (0.2, 1, 5, 20, and 50 μg/mL) were injected in order to determine the standards.

### 4.5. Ascorbic Acid Determination (AsA)

The assessment of ascorbic acid (AsA) and dehydroascorbic acid (DAsA) amount was performed using the HPLC method, as described by Brubacher [34,52]. As for the DAsA reduction process, a method in accordance with Ohta and Harada was conducted [34,53]. The solutions of lyophilized juice and extract in the quantity of 10 mg were made using BCLJ and BCLW, with 1 mL of 4.5% metaphosphoric acid. An ultrasonic bath was used in order to dissolve the samples. After dissolution within 10 min and centrifugation for 15 min at 10,000× *g* rpm, the supernatant was separated and used for further analysis. In this way, AsA was evaluated. After addition of 1 mL of the reducing agent (50 μM 1,4-dithiothreitol—DTT) to 1 mL of the sample, DAsA was evaluated. The prepared solution was held at a room temperature for 10 min, due to the chemical reaction. An amount of 20 μL of the filtered sample was injected into the HPLC. The obtained results were noted as μg/100 mg of BCLJ and BCWE as a quantity of total ascorbic acid (TAsA = AsA + DAsA).

#### Chromatographic Conditions

For the separation, as well as the quantification of AC and the determination of AsA, one system was used. Phosphate buffer (40 mM) and methanol were the mobile phase. The ratio was 92:8 under isocratic conditions. Chromatographic flow rate was 0.8 mL/min at room temperature, whilst the absorbance was measured at 244 nm. In order to construct the calibration curve, triple standards of AsA (1, 10, 50, 100, 150, and 200 μg/mL) were injected for every single concentration.

### 4.6. Determination of Flavonol Content

The hydrolysis of flavonols was essential for the sample preparation. Ten milligrams of the extract was dissolved in 1 mL of the mixed methanol and hydrochloric acid (1:1, V/V). An ultrasonic bath was used to dissolve the samples, which lasted for 10 min. Afterwards, the samples were placed in a water bath at a temperature of 90 °C for 15 min. The samples were centrifuged after the water bath for 20 min at 4 °C with a rotation rate of 10,000× *g* rpm. After filtration, the supernatants were used for further HPLC analysis [34,54].

#### Chromatographic Conditions

The abovementioned chromatographic system for analysis of ACs and ASA was also used for flavonols. For acidification, trifluoroacetic acid 0.1% was used as mobile phase A, whilst mobile phase B was acetonitrile. Flavonoid separation was performed by gradient: 85% (A) from 0 to 20 min; 65% (A) from 20 to 24 min; 50% (A) from 24 to 29 min; then 10% (A) to 31 min; and 15% for the upcoming 2 min. The amount of the sample was 10 μL, and the flow rate was 0.7 mL/min. The column temperature was 30 °C, and the absorbance was measured at 360 nm. Determination of the flavonol contents was performed using the calibration curves. To construct the curves, flavonol standards—myricetin, quercetin, and kaempferol—were used. The quantity of flavonols was presented as μg of flavonols per 100 mg of the lyophilized extract.

The quantification of phenolic compounds was carried out using standard calibration curves. These curves were established over concentration ranges of 0.2–50 mg/L for anthocyanins and 0.2–50 mg/L for flavonols. The coefficients of determination (R^2^), limits of detection (LOD), and limits of quantification (LOQ) are presented in Table 3. The LOD and LOQ were calculated using the following equations:

LOD = 3S_a_/b

LOQ = 10S_a_/b

In these equations, S_a_ represents the standard deviation of the response and b denotes the slope of the calibration curve.

### 4.7. 2,2-Diphenyl-1-picrylhydrazyl (DPPH) Radical Scavenging Activity

The DPPH method was performed to investigate radical scavenging activity in compliance with the Konić-Ristić method, with minor updates [34,55]. Total amounts of 3 mg lyophilized juice and waste were dissolved in 1 mL of distilled water and ethanol, respectively. Afterwards, the samples were treated in an ultrasonic bath for 5 min. Centrifugation lasted 10 min at 2500× *g* rpm. The obtained supernatant was used for further analysis. Seven receding concentrations were obtained by dilution. Methanol (120 μL) and DPPH reagent (40 μL; 0.05 mM) were added to every sample. They were left in a microtiter plate and in the shade for 30 min, when the peak of the reaction occurred. The optical density of the samples was measured on an enzyme-linked immunosorbent assay (ELISA) reader (Multiskan Ascent Thermolabsystems Elisa No 354, Thermo Fisher Scientific, Vantaa, Finland), per wavelength of 540 nm. The percentage of free DPPH radical inhibition (%) was determined per the formula:

% inhibition = (Ac − As/Ac − AB) × 100

In the equation, AB represents the absorbance of the blank test (the solvent); Ac represents the absorbance of the control (mixture of the solvent and DPPH); and As is the absorbance of the sample. Methanol was used as a blank. The positive control comprised ascorbic acid, butylhydroxy anisole (BHA), butylhydroxy toluene (BHT), and Trolox (6-hydroxy-2,5,7,8-tetramethylchroman-2-carboxylic acid). The results were presented as the sample concentration needed to inhibit 50% of the free DPPH of radicals (IC50). For this purpose, calculation is performed from the line equation. The equation presents the ratios of sample concentrations and percentage of inhibition.

### 4.8. β-Carotene Bleaching Method

The β-carotene bleaching method was performed in alignment with spectrophotometric measurements [34,56]. Antioxidant potential of the sample was measured by the capability of the sample to prevent oxidative loss of β-carotene in the β-carotene/linoleic acid emulsion. In order to prepare the emulsion, 2 mg of crystalline β-carotene was dissolved in 10 mL of chloroform. Linoleic acid at a volume of 25 μL and 180 mg of the emulsifier Tween 20 were poured into 1 mL of the obtained solution. Chloroform was evaporated using a vacuum evaporator at 40 °C. After that, 50 mL of oxygenated water was added to the system. This emulsion was shaken until it became limpid. Emulsion at a volume of 0.16 mL was transferred into the wells of the microtiter plates. These wells contained the previously added dilution series of blackcurrant juices or extracts at a volume of 0.04 mL. The microtiter plates were then mixed on a mixer. Afterwards, the initial absorbance (A0) was analyzed on an ELISA reader at a wavelength of 450 nm. The microtiter plates were incubated at 55 °C for two hours. Absorbance (A2h) was noted again. The antioxidative activity was determined in alignment with the following equation:

% inhibition = (A2h/A0) × 100

Ascorbic acid, BHA, BHT, and Trolox acid were used as positive controls. The β-carotene bleaching method results were noted as the sample concentration required to inhibit the toll of 50% of the β-carotene (IC50). Similar to previous methods, percentage of inhibition was calculated from the ratio of concentration and percentage of inhibition.

### 4.9. Animals and Experimental Design

For this study, 30 healthy male Wistar albino rats (250–320 g) were used, allocated randomly into five groups (n = 6). The experiment was conducted at the Vivarium of the Scientific Research Center for Biomedicine, Faculty of Medicine, University of Niš, Serbia, under strictly controlled environmental conditions (12 h light/dark cycle, 20 ± 2 °C, polycarbonate cages, commercial food and water ad libitum). The protocol was approved by the Ethics Committee of the Ministry of Agriculture, Forestry and Water Management—Veterinary Administration (decision no. 327-07-09101/2020-05/2) and complied with the EU Directive 2010/63/EU on animal experiments.

Following a 7-day adaptation period, groups were assigned as follows: Control group: 1 mL saline daily; BCLW1, BCLW2, BCLW3: (100, 200, 300 mg/kg b.w.); BCLJ (200 mg/kg b.w.). Substances were administered orally via intragastric tube once daily for 10 days. Lyophilized extracts were reconstituted in water immediately before administration.

A pilot study was first performed to optimize conditions and dosage, ensuring safety, minimal stress, stability, and solubility of preparations, thereby validating the design for the main experiment, according to the experiment by Milutinović et al. (2022) [57]. The primary phase included a small number of rats and helped determine the experimental conditions. The information from the pilot experiment ensured that the data were valid and reliable on a larger number of animals. Multiple circumstances were considered during the specification of the dosage. These included animal safety, well-being, and minimal stress during administration. Another important factor was to maintain the stability and solubility of the black currant preparations. Also, no unnecessary stress was induced and normal activity was not disrupted.

#### 4.9.1. Sample Collection

On the eleventh day of the experiment, the rats were sacrificed with an anesthetic (Ketamidor 10%, Richter Pharma AG, Wels, Austria). Afterwards, 2 mL of blood was taken from the aorta for biochemical analyses. Also, plasma was separated using centrifugation at 4000× *g* rpm, which lasted 20 min. The kidneys were taken out and rinsed with ice-cold saline. For histopathological examination, the kidneys were isolated and sectioned along the sagittal plane, with care taken to ensure that the papilla remained in one of the two half-kidneys, and promptly fixed in 10% buffered formaldehyde for 24 h.

#### 4.9.2. Histopathological and Immunohistochemical Analyses of Kidney Tissue

Half of each kidney was promptly fixed in 10% phosphate-buffered formaldehyde for 24 h. Tissue processing for paraffin embedding was performed in an automated tissue processor (Myr STP120; Myr, Tarragona, Spain) using standard paraffin-processing steps: graded ethanol dehydration, xylene clearing, and paraffin wax infiltration. Dehydration was performed at ascending ethanol concentrations (70%, 90%, and 100%, with 100% repeated as needed), followed by clearing in xylene.

For paraffin impregnation, tissues were infiltrated with histology-grade paraffin wax. Paraffin blocks were sectioned at 4 μm using a rotary microtome (Leica RM2235 rotary microtome (Leica Biosystems, Nuβloch, Germany)), and sections were mounted on glass slides and dried prior to staining. Nuclear staining was performed with hematoxylin (0.2% *w*/*v*). Counterstaining was performed with eosin Y (0.5% *w*/*v*) [58].

For immunohistochemical analysis, antigen retrieval was performed by pretreating tissue sections with 10 mM citrate buffer in a microwave oven for 10 min. Rabbit monoclonal primary antibody against Ki67 (clone 30-9) was used in a Ventana BenchMark GX instrument (Roche, Tucson, AZ, USA), with the BMK ultraView DAB procedure. Sections were then dehydrated in ascending ethanol series (70%, 90%, and 100%), clarified with xylene and mounted with DPX mount (Sigma-Aldrich, St. Louis, MO, USA). Negative controls included buffer alone. Histological and immunohistochemical analysis was performed in polyclinics Poliklinika Human (Niš).

#### 4.9.3. Image Analysis

The images were obtained using a 40× objective (NA = 0.65) of a CX43 microscope (Olympus, Tokyo, Japan). These digital images of 24 M pixels were obtained by Nikon D5300 digital camera (Nikon, Tokyo, Japan).

The Ki67 index (the percentage of positively stained cell nuclei) was estimated manually with ImageJ 1.53s software, multi-point tool (Wayne Rasband, National Institutes of Health (Bethesda, MD, USA), in cortical proximal tubules, proximal tubules of distal medulla (S3 segment), collecting ducts, and urothelial cells of the renal pelvis. At least 200 nuclei were counted per each animal tissue.

### 4.10. Statistical Analysis

The obtained results for TP, TT, ACs, flavonols, AsA, and AO activity were processed and analyzed using the SPSS statistical software package (version 25.0; Chicago, IL, USA).

Each value was presented as mean value ± standard deviation (SD). This was due to the fact that all measurements were conducted in triplicate. Regression analysis was used in order to obtain the EC50 values (the concentration resulting in 50% of the maximum response). Statistically significant differences among the EC50 values of BCLJ and BCLW were noted using Student’s *t*-test for independent samples in case the distribution was normal. Also, one-way ANOVA (analysis of variance) test was used for evaluation of variables between multiple groups, for normal distribution, together with Duncan’s post hoc test (*p* < 0.05 or *p* < 0.01).

## 5. Conclusions

The present study demonstrates that lyophilized black currant juice (BCLJ) and black currant waste extract (BCLW) from the *Ribes nigrum* L. variety Čačanska crna are rich sources of bioactive compounds, particularly anthocyanins, flavonols, polyphenols, and ascorbic acid. Both preparations exhibited notable antioxidant activity in vitro, with BCLW showing superior radical scavenging capacity, likely due to its higher content of delphinidin- and cyanidin-based anthocyanins, especially rutinosides.

In vivo evaluation revealed that administration of black currant extracts led to a reduction in the Ki67 index in adult rat kidney epithelial cells, most prominently in the collecting ducts and proximal tubules following treatment with BCLW. Given that adult kidney tissue is characterized by intrinsically low proliferative activity, the observed decrease in Ki67 expression suggests that black currant extracts do not induce proliferative stress in healthy renal tissue and may contribute to the maintenance of cellular quiescence under physiological conditions.

Overall, the results indicate that black currant juice and waste extracts possess strong antioxidant potential and exert antiproliferative effects without evidence of adverse stimulation of cell proliferation in normal kidney tissue. These findings provide novel in vivo data on the interaction between anthocyanin-rich black currant preparations and healthy renal epithelium and support their potential role as safe functional food components. Further studies incorporating functional renal markers, oxidative stress parameters, and injury models are warranted to clarify the biological significance and possible preventive implications of these effects.

## Figures and Tables

**Table 1:** Chemical composition of BCLJ and BCLW.

Compounds	Units	BCLJ	BCLW
Total polyphenols	mg GAE/g	6.97 ± 0.54 ^a^	4.54 ± 0.38 ^b^
Total tannins		4.19 ± 0.36 ^a^	3.98 ± 0.44 ^a^
D3G	μg/100 mg	107.34 ± 4.40 ^a^	271.1 ± 3.81 ^b^
D3R		394.92 ± 15.92 ^a^	757.78 ± 27.12 ^b^
C3G		34.16 ± 1.15 ^a^	68.65 ± 5.21 ^b^
C3R		271.13 ± 10.17 ^a^	464.35 ± 12.47 ^b^
TAsA		750.00 ± 15.14 ^a^	50.00 ± 3.64 ^b^
Myricetin		83.80 ± 6.61 ^a^	5.79 ± 0.95 ^b^
Quercetin		22.10 ± 1.76 ^a^	1.13 ± 0.06 ^b^
Kaempferol		2.70 ± 0.05 ^a^	0.7 ± 0.00 ^b^

The values in the table are the means of three replicate determinations (n = 3) ± standard deviations, D3G—delphinidin-3-*O*-rutinoside, D3G—delphinidin-3-*O*-glucoside, C3G—cyanidin-3-*O*-glucoside and C3R—cyanidin-3-*O*-rutinoside, TAsA—total ascorbic acid. Different letters in the row (^a,b^) represent a statistically significant difference according to Student’s *t*-test at *p* < 0.05.

**Table 2:** Antioxidative capacity of BCLJ and BCLW in mg/mL evaluated by 2,2-diphenyl-1-picrylhydrazyl (DPPH) radical scavenging and *β*-carotene/linoleic acid systems.

Sample	DPPH (mg/mL)	*β*-Carotene (mg/mL)
BCLJ	4.89 ± 0.26 ^a^	12.54 ± 1.39 ^a^
BCLW	3.97 ± 0.18 ^b^	1.32 ± 0.31 ^b^

The values represent mean values of three measurements (n = 3) ± standard deviations. Different letters in the columns (^a,b^) indicate a statistically significant difference according to Student’s *t*-test at *p* < 0.05.

**Table 3:** Phenolic compounds quantified by HPLC with determination coefficient (R^2^), limits of detection (LOD), and quantification (LOQ).

Phenolic Compounds	R^2^	LOD	LOQ
Anthocyanins			
D3R	0.9990	3.20	9.54
D3G	0.9988	1.75	5.78
C3R	0.9992	4.50	18.24
C3G	0.9935	1.22	4.12
Flavonols			
M	0.9991	0.25	0.80
Q	0.9976	0.16	0.9
K	0.9999	0.20	0.55

D3R—Delphinidin-3-O-rutinoside; D3G—Delphinidin-3-O-glucoside; C3R—Cyanidin-3-O-rutinoside; C3G—Cyanidin-3-O-glucoside: M—Myricetin; Q—Quercetin; K—Kaempferol.

## Data Availability

Data are contained within the article.
